# SARS-CoV-2 Infections, Re-Infections and Clinical Characteristics: A Two-Year Retrospective Study in a Large University Hospital Cohort of Vaccinated Healthcare Workers

**DOI:** 10.3390/jcm12216800

**Published:** 2023-10-27

**Authors:** Luigi De Maria, Giuseppe Delvecchio, Stefania Sponselli, Francesco Cafaro, Antonio Caputi, Gianmarco Giannelli, Pasquale Stefanizzi, Francesco Paolo Bianchi, Angela Stufano, Silvio Tafuri, Piero Lovreglio, Paolo Boffetta, Luigi Vimercati

**Affiliations:** 1 Interdisciplinary Department of Medicine, University of Bari, 70124 Bari, Italy; luigi.demaria@uniba.it (L.D.M.); giuseppe.delvecchio1@uniba.it (G.D.); stefania.sponselli@uniba.it (S.S.); francesco.cafaro@uniba.it (F.C.); gianmarco.giannelli@uniba.it (G.G.); pasquale.stefanizzi@uniba.it (P.S.); angela.stufano@uniba.it (A.S.); silvio.tafuri@uniba.it (S.T.); piero.lovreglio@uniba.it (P.L.); luigi.vimercati@uniba.it (L.V.); 2 Health Prevention Department, Local Health Authority of Brindisi, 72100 Brindisi, Italy; francescopaolo.bianchi@asl.brindisi.it; 3 Stony Brook Cancer Center, Stony Brook University, Stony Brook, NY 11794, USA; paolo.boffetta@unibo.it; 4 Department of Medical and Surgical Sciences, University of Bologna, 40138 Bologna, Italy

**Keywords:** COVID-19, SARS-CoV-2 breakthrough infections, healthcare workers

## Abstract

At the University Hospital of Bari, during the first year after the start of the mandatory vaccination campaign with BNT162b2 mRNA COVID-19 vaccine, the preliminary results of an observational study showed a significant prevalence of SARS-CoV-2 breakthrough infections (BIs) among healthcare workers (HCWs), but no hospitalization or deaths. In the present study, we extended the observation period (January 2021–January 2023) with the aim of determining the incidence, characteristics and clinical course of SARS-CoV-2 BIs among 6213 HCWs. All HCWs were regularly monitored and screened. To allow return to work after BI, the protocol required one negative nasopharyngeal swab test followed by a medical examination certifying complete clinical recovery. We observed an overall incidence rate of SARS-CoV-2 BIs of 20.2%. Females were most affected, especially in the nurse group compared with doctors and other HCWs (*p* < 0.0001). Cardiovascular diseases were the most frequent comorbidity (*n* = 140; 11.4%). The source of infection was non-occupational in 52.4% of cases. Most cases (96.9%) showed minor symptoms and only two cases of hospitalization (one in intensive care unit), 13 cases of re-infection and no deaths were recorded. Our results confirm that SARS-CoV-2 infection can break vaccination protection but the clinical course is favorable.

## 1. Introduction

Since the start of the SARS-CoV-2 pandemic, many countries have experienced multiple waves of virus outbreaks. Italy was one of the first affected countries, with more than 25 million confirmed cases and about 190,000 deaths [1,2]. Health care workers (HCWs) are one of the most exposed categories at risk of infection [3,4,5]. Since February 2020, about 485 thousand cases of COVID-19 have been diagnosed among Italian HCWs, and it has been widely demonstrated that preserving the health and well-being of HCWs is critical to the maintenance and integrity of health systems and the quality of patient care [6,7,8].

The transmission mechanisms of SARS-CoV-2 have changed during the pandemic thanks to vaccination, natural infections and the emergence of new variants [9,10,11,12]. As of 30 July 2023, in Italy, 90% of the vaccinated subjects over 12 years old have completed the vaccination course [13]. Several studies and meta-analyses have been conducted on the effectiveness of vaccination in occupational settings, showing a substantial reduction in infections, hospitalizations and deaths [14,15]. A recent retrospective cohort study of 10,024 breakthrough infections (BIs) showed how receiving at least one COVID-19 vaccine dose was associated with a significantly lower risk of respiratory failure, intensive care unit admission, intubation/ventilation, hypoxaemia and oxygen requirement, but not other outcomes (including long-COVID features), while receiving two vaccine doses was associated with lower risks for most outcomes [16].

Nevertheless, SARS-CoV-2 breakthrough infections in vaccinated subjects and re-infections in previously infected individuals have become increasingly widespread, even in high-risk occupational settings, such as healthcare [15,17,18]. One explanation for this phenomenon is that the emergence of SARS-CoV-2 variants of concern (VOC) limits the success of vaccines and natural immunity, as they contain genomic alterations, particularly in the spike protein coding regions [19]. The vast majority of vaccines targeted the SARS-CoV-2 spike protein as a key antigen, based on the virus lineage originally identified in Wuhan [20]. The B.1.1.7 (Alpha) variant was first identified from genomic sequencing of samples obtained from COVID-19 patients and was responsible for an increasing number of cases in England in late 2020. Afterwards, the emergence of the B.1.351 (Beta) variant in South Africa and the P.1 (Gamma) variant in Brazil expanded the COVID-19 pandemic. At the end of 2020, a novel SARS-CoV-2 variant, the B.1.617.2 (Delta) variant was first discovered, determining a strong increase in cases of COVID-19 and deaths in India and nearby countries. In December 2021, the B.1.1.529 (Omicron) variant emerged. It contained about 30 mutations in the spike protein, threatening the immune defenses acquired naturally and through vaccination [21].

Continuing to study and understand the transmission mechanisms of SARS-CoV-2 variants and the impact of vaccination and previous infection is particularly important in vulnerable and high-risk populations, such as those in healthcare settings, characterized by the presence of fragile patients. This topic, to date, also deserves renewed attention due to the worldwide reduction in restrictive policies and preventive measures, such as the mandatory use of personal protective equipment (PPE), social distancing and staggered access to hospital wards following the end of the pandemic state of emergency. All these preventive measures had proven effective in curbing the spread of the virus during the pandemic phase [22,23,24].

At the University Hospital of Bari, one of the largest COVID-19 hub centers in southern Italy, during the first year after the start of the widespread mandatory vaccination campaign for all HCWs, an observational study has been set [25]. Preliminary results in a limited observation time window (13 months) showed a significant prevalence (9.7%) of SARS-CoV-2 BIs, while no cases of long-COVID and no hospitalization or deaths were recorded.

In the present study, we extended the observation period to January 2023 (24 months) with the aim of determining the incidence, characteristics and clinical course of SARS-CoV-2 BIs among HCWs at a longer time distance from the completion of vaccination cycle.

## 2. Materials and Methods

### 2.1. Study Design, Setting, Population

We set a retrospective observational study on 6213 HCWs, covering a two-year period (January 2021–January 2023). HCWs cohort was part of the European Commission-sponsored Orchestra project. The vaccination campaign with the BNT162b2 mRNA COVID-19 vaccine started on 27 December 2020.

During the study period, all HCWs were regularly monitored and subjected to the preventive protocol established by the Operative Unit of Occupational Medicine. According to the protocol, study subjects were screened with nasopharyngeal RT-PCR swabs every fourteen days. Quick access to molecular testing was provided for close contacts with COVID-19 cases and symptomatic subjects. HCWs also had to undergo a health surveillance medical examination at the Operative Unit of Occupational Medicine before returning to work after contracting COVID-19. Additional laboratory tests, instrumental tests and specialist evaluations were part of the health surveillance and performed if necessary. Return to work after COVID-19 was allowed only if HCWs tested negative on a nasopharyngeal RT-PCR or antigenic swab and in the absence of signs and symptoms of the disease.

The SARS-CoV-2 BIs are defined as the detection of virus RNA or antigen in the respiratory samples of an individual 14 days after the receipt of a second dose of COVID-19 vaccine [26].

We classified HCWs into three occupational categories: doctors, nurses and other HCWs (i.e., biologists, technicians, administrative staff, psychologists). The hospital’s operating units were stratified into “COVID-19 low risk units” (CLRUs) and “COVID-19 high risk units” (CHRUs) according to the assessment of biological risk from exposure to SARS-CoV-2. CHRUs are operating units with a high frequency of high-risk procedures such as direct assistance to COVID-19 patients, invasive maneuvers with aerosol generation, and handling of potentially infected biological samples.

For all cases of breakthrough infection, the following characteristics were examined: gender, age, smoking habit vaccination status, source of the infection and comorbidities (diabetes mellitus, cardiovascular diseases, respiratory diseases, immunodeficiency disorders, solid or hematologic malignancies). Details of comorbidities are shown in Table 1.

The clinical course was evaluated by considering the duration of the infection, symptoms (duration and persistence after negative swab) and outcomes such as hospitalizations and deaths. Symptoms were categorized into major and minor. “Major symptoms” included altered state of consciousness, dyspnea, inability to walk, lymphadenopathy, bleeding, anxiety and depression, memory loss, anorexia, aphasia, syncopal episodes, and epileptic seizures. “Minor symptoms” included fever, abdominal pain, vomiting, nausea, dysgeusia, ageusia, loss of smell, cough, chest pain, diarrhea, weight loss, fatigue, headache, myalgias, rhinorrhea, sore throat, conjunctivitis, skin rash, dizziness, insomnia, and concentration disorders.

All study participants were informed that data from the research protocol would be treated in an anonymous and collective way, according to scientific methods and for scientific purposes, in agreement with the principles of the Declaration of Helsinki. Ethical approval was not necessary because all medical and instrumental examinations were performed according to Italian law concerning the protection of workers exposed to occupational risks (D.Lgs. 81/2008). Nevertheless, the study was approved by the Ethics Committee of Azienda Ospedaliero-Universitaria Consorziale Policlinico of Bari (Parere Studio N. 7241).

### 2.2. Statistical Analysis

The analysis was performed using Stata MP18 software. Continuous variables were expressed as mean ± standard deviation and range, and categorical variables as proportions. The normality of the continuous variables was evaluated using the test of Skeweness and kurtosis, but none resulted were normally distributed and it was not possible to build normalization models. Thus, continuous variables were compared between two groups using the Wilcoxon rank-sum test and between multiple groups using the Kruskal–Wallis test; categorical variables were compared between groups using the chi-square test or Fisher’s exact test. For all tests, a *p*-value < 0.05 was considered statistically significant.

## 3. Results

### 3.1. Breakthrough Infections: Frequency and Characteristics

A total of 98% of 6234 HCWs completed the vaccination cycle. Among these, we observed 1234 cases of breakthrough infections: 809 (65.6%) female and 425 (34.4%) male. The overall incidence rate of SARS-CoV-2 BIs was 20.2%. Most of the cases were observed among doctors (*n* = 455; 36.9%) and nurses (*n* = 446; 36.1%), who were almost equally affected. The characteristics of the BI cases, grouped by occupational categories, are described in Table 2 and Table 3.

Females were most affected, especially in the nurse group compared with doctors and other HCWs (*p* < 0.0001). Doctors with BI were also significantly younger (average age = 37.9 years) than nurses (average age = 43.6 years) and others (average age = 50.4 years). The prevalence of non-smokers was high in all occupational categories (68.2%), particularly among doctors (76.7%; *p* < 0.0001), who also showed a lower BMI (average BMI = 23.3 ± 3.6; *p* < 0.001). Cardiovascular diseases were the most frequent comorbidity (*n* = 140; 11.4%), followed by immunodeficiency disorders (*n* = 69; 5.6%), respiratory diseases (*n* = 44; 3.6%), diabetes mellitus (*n* = 22; 1.8%) and solid or hematologic malignancies (*n* = 17; 1.4%). Only 15.5% of BIs affected HCWs working in CHRUs and, among these, nurses (*n* = 97; 21.8%) and doctors (*n* = 74; 16.3%) were significantly more affected compared to other HCWs (*p* < 0.0001). In total, 95.7% of BIs were diagnosed after the third vaccine dose, and only 4.3% after the second with no significant differences between the different occupational categories (*p* = 0.100). The infection lasted an average of 11 days (10.9 ± 3.9; range 5–41); the source of infection was non-occupational in 52.4% of cases and occupational in 22.2% of cases (12.7% contagion from colleagues and 9.5% contagion from patients). In total, 63.2% of BIs were diagnosed following a nasopharyngeal swab carried out for symptoms, while 36.1% for screening in asymptomatic HCWs.

**Figure 1:**
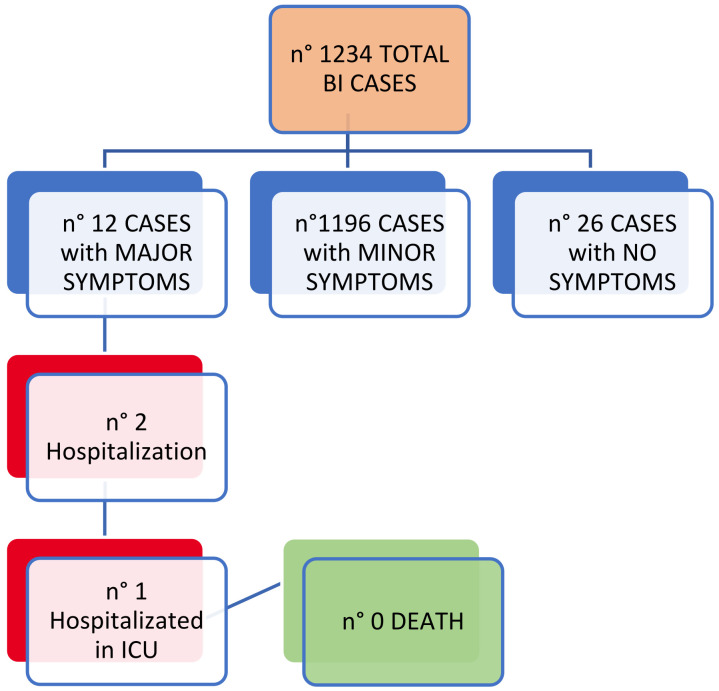
BIs: clinical outcomes.

Most cases (96.9%) of BIs showed minor symptoms of average duration of 9 days (9.2 ± 5.1; range 0–46) and only 12 cases (1%) showed major symptoms. Of these symptomatic BIs, 176 cases (14.3%) continued to show symptoms after the negative swab at the end of the infection, and in three cases (1.7%) the symptoms continued for at least or more than 15 days. We also observed two hospitalizations, one of which in the intensive care unit (Figure 1). No deaths were recorded.

A comparison of BIs characteristics between CHRUs and CLRUs (Table 4) showed no significant differences in vaccination status, duration of infection, symptoms, clinical course and outcomes (hospitalizations, admissions to intensive care unit, duration of symptoms after recovery from infection), while the frequency of non-occupational source of infection was significantly higher in CHRUs than in CLRUs (62.3% vs. 50.6%; *p* = 0.012).

We observed 13 re-infections (1.1%) with a mean distance from the first infection of 263.6 ± 123.2 days (range: 81–563); none worked in the CHRUs, nor were there any cases of persistence of symptoms after recovery from the infection or hospitalizations. The source of the infection was not known in seven (54%) cases, while in three (23%) cases was non-occupational and in three (23%) cases occupational: two (15.4%) from patients and one (7.6%) from colleagues. The nasopharyngeal swab was performed in 10 (76.9%) cases for symptoms and in three (23.1%) for screening. The infection was asymptomatic in two (14.4%) cases, minor symptoms were shown in 11 (84.6%) cases and the duration of symptoms was on average 4.8 ± 2.7 days (0–11).

### 3.2. Description of Hospitalization Cases

Of the two hospitalized HCWs, the first was a 24-year-old nurse with a full vaccination cycle (two doses). She was admitted to the COVID-19 ward showing fever and cough. A chest X-ray revealed minor consolidation in the lower right lung region. After receiving treatment with ceftriaxone and enoxaparin, she recovered and was discharged ten days later, after a negative nasopharyngeal swab test, with no signs or symptoms of the disease. The other hospitalized HCW was a 54-year-old doctor with a complete vaccination cycle (three doses). He was admitted to the COVID-19 intensive care unit with high fever, rest dyspnea, diarrhea, ageusia, anosmia, myalgia, asthenia and pharyngodynia. During the hospitalization ECG, blood chemistry analysis, chest CT scan, blood culture and Doppler ultrasound of the lower extremities were conducted, all of which showed no significant alterations. He received successful treatment with amoxicillin, vitamins C and D, probiotics, and corticosteroids, and was discharged a month later with persistence of only minor respiratory symptoms. At the time of the medical examination to return to work, due to the still persistent minor respiratory symptoms, further consultations with pneumology and cardiology specialists were conducted, all of which resulted negative for significant pathological findings.

## 4. Discussion

In this study, we aimed to evaluate the incidence, characteristics and clinical course of COVID-19 BIs among HCWs of the University Hospital of Bari, during a two-year observation period. We found an overall incidence rate of SARS-CoV-2 BIs of 20.2%. Considering that this rate was 9.7% in February 2022, the increase in BIs over the last 11 months was remarkable [25]. Furthermore, this data is also significantly higher when compared to the frequency of BIs cases (9%) in the first three waves of the pandemic during the pre-vaccination era, among the same population of HCWs [22]. The literature data report a lower incidence of BIs in fully vaccinated HCWs [27,28]. A recent systematic review and meta-analysis on post-vaccination SARS-CoV-2 infection among HCWs showed that, among fully vaccinated HCWs, across 16 studies, the overall pooled proportion of COVID-19 infections was 1.3% (95% CI 0.6–2.9; I2 99.3%) [29]. Considering that, in our study, the source of the infection was non-occupational in 52.4% of cases, while occupational only in 22.2% of cases, our hypothesis is that the high frequency of BIs found may be due to the progressive easing of government restrictive measures among the general population as well as to a progressive failure to comply with correct good hygiene practices induced by the perception of protection generated by the availability of vaccines. Moreover, the role of new variants should not be underestimated. VOC may penetrate herd immunity and facilitate vaccine escape, which can predispose these individuals to severe disease or death. In a recent meta-analysis including eleven randomized controlled trials (161,388 participants), 20 cohort studies (52,782,321 participants), and 26 case–control studies (2,584,732 cases), Baoqi Zeng et al. aimed to provide a comprehensive overview of the effectiveness profile of COVID-19 vaccines against VOC. Full vaccination was effective against Alpha, Beta, Gamma, Delta, and Omicron variants, with vaccine effectiveness (VE) of 88.0% (95% CI, 83.0–91.5), 73.0% (95% CI, 64.3–79.5), 63.0% (95% CI, 47.9–73.7), 77.8% (95% CI, 72.7–82.0), and 55.9% (95% CI, 40.9–67.0), respectively. Booster vaccination was more effective against Delta and Omicron variants, with VE of 95.5% (95% CI, 94.2–96.5) and 80.8% (95% CI, 58.6–91.1), respectively. mRNA vaccines (mRNA-1273/BNT162b2) seemed to have higher VE against VOC over others; significant interactions were observed between VE and vaccine type (mRNA vaccines vs. not mRNA vaccines) [30]. Other authors investigated the effects of SARS-CoV-2 virus type and of vaccination status on causes of death. During the observation period, 234 patients died in the context of their inpatient stay, with a positive PCR test for SARS-CoV-2-RNA. A total of 117 deceased patients were infected with wild-type SARS-CoV-2, 33 with the alpha variant, 38 with the delta variant, and 19 with the omicron subtype. The rate of patients who died from SARS-CoV-2 infection was as follows for the individual virus subtypes: 85% (wild type), 94% (alpha), 82% delta, and 46% (omicron). Of these, 24% of patients infected with the delta subtype had been vaccinated/received a booster vaccine, of which 16% experienced a severe course of COVID-19 disease. Of those patients infected with the Omicron strain, the proportion of deaths in patients who had been vaccinated/received a booster was 41%, in all of whom COVID-19 took a severe course [31].

Furthermore, in our study, as many as 36.1% of cases were diagnosed via nasopharyngeal swab carried out for screening, confirming the fundamental role of this preventive procedure in intercepting asymptomatic cases and reducing viral circulation, as it has been since the pre-vaccination phase [32,33,34].

As expected, females were more affected than males. This data is in agreement with the gender differences shown in several studies and, also, with national-level findings since the pre-vaccination phase [6,25,28,35].

Moreover, in agreement with the literature data, HCWs with patient-facing clinical tasks (doctors and nurses) were the most affected professional category [36]. Only 15.5% of BIs affected HCWs working in CHRUs; as already discussed in the previous study, this result can be explained by the highest attention to prevention and protection measures observed by healthcare workers engaged in operational units at high risk of contagion [25].

In agreement with literature reviews and meta-analyses on risk factors and comorbidities of SARS-CoV-2 infection, in our study cardiovascular diseases were the most frequent comorbidity in BI cases (*n* = 140; 11.4%), followed by immunodeficiency disorders (*n* = 69; 5.6%), respiratory diseases (*n* = 44; 3.6%), diabetes mellitus (*n* = 22; 1.8%) and solid or hematologic malignancies (*n* = 17; 1.4%) [37,38]. A possible interpretation is that diseases such as cardiovascular disease, diabetes and respiratory disease may share certain pathogenetic mechanisms with COVID-19, such as the proinflammatory state or the attenuation of the innate immune response. In particular, diabetes is one of the recognized risk factors associated with mortality triggered by COVID-19, and is strictly characterized by compromised immunity because of the accumulation of activated innate immune cells in metabolic tissues that leads to the release of inflammatory IL-1β and TNFα, which promote systemic insulin resistance and β-cell damage. This process is presumed to lead to increased susceptibility to COVID-19, particularly in those with high blood glucose [39].

Finally, in this study, the analysis of clinical outcomes strengthens the good results already found in the past in the same cohort and confirmed by the literature data [25,40]. In particular, we observed only two hospitalizations, one of which in the intensive care unit and no deaths. A recent study by Tenforde et al. demonstrated that 84.2% of hospitalizations occurred in unvaccinated subjects [41]. Also, disease progression to intensive care unit recovery or death was associated with a decreased odd of vaccination. Other authors showed that vaccination was associated with reduced probability of hospitalization [42]. In addition, vaccines may attenuate disease severity. In our study, most cases (96.9%) of BIs showed minor symptoms of average duration of 9 days, and only 12 cases (1%) showed major symptoms. Of these symptomatic BIs, 176 cases (14.3%) continued to show symptoms after the negative swab at the end of the infection, and in only three cases (1.7%) did the symptoms continue for at least or more than 15 days. In a case series of SARS-CoV-2 BIs among HCWs, Alshamrani et al. found that most of HCWs had mild (52.6%) or moderate (10.3%) disease with no need for hospitalization [43].

The main limitations of the study lie in the absence of the analysis of the virus variants and correlation with the antibody titer, which would have required unavailable resources.

Nevertheless, the large cohort of HCWs carefully subjected to health surveillance and followed for a long period through close clinical monitoring are the strengths of this study, which can thus contribute to the international debate about the need for vaccination in HCWs and its effectiveness against SARS-CoV-2.

## 5. Conclusions

In conclusion, despite the high incidence rate of SARS-CoV-2 infection in vaccinated HCWs observed in our two-year cohort study (20.2%), the clinical course was favorable: most cases showed minor symptoms, cardiovascular diseases were the most frequent comorbidity, and only two cases of hospitalization (one in intensive care unit), 13 cases of re-infection, and no deaths were recorded. The BNT162b2 vaccination seemed to provide good protection from severe disease, but lower protection from transmission; therefore, it is necessary to continue to focus with great attention on prevention and protection measures from biological risk, particularly in hospital settings at high risk for the health of HCWs and fragile patients.

## Figures and Tables

**Table 1:** Comorbidities.

Cardiovascular Diseases	Respiratory Diseases	Immunodeficiency Disorders
ischemic cardiomyopathy	allergic bronchial asthma	drug related
ischemic stroke	chronic obstructive pulmonary disease	congenital
cardiac arrhythmia	obstructive sleep apnea syndrome	chronic disorders
arterial hypertension		autoimmune
chronic heart failure NYHA I-IV		transplants

**Table 2:** Characteristics of the BI cases by occupational categories: gender, age, smoking habit and comorbidities.

	Nurses (*n* = 446)	Doctors (*n* = 455)	Other HCWs (*n* = 333)	Total (*n* = 1234)	*p*-Value
Female; *n* (%)	327 (73.3)	268 (58.9)	214 (64.3)	809 (65.6)	<0.0001
Male; *n* (%)	119 (26.7)	187 (41.1)	119 (35.7)	425 (34.4)	
Age (years); average ± SD (range)	43.6 ± 11.3 (24–66)	37.9 ± 10.7 (26–70)	50.4 ± 12.0 (20–69)	43.4 ± 12.3 (20–70)	<0.001
Smoking habit; *n* (%) Non smoker Smoker Ex-smoker	287 (64.3) 95 (21.4) 64 (14.4)	349 (76.7) 71 (15.6) 35 (7.7)	206 (61.9) 97 (29.1) 30 (9.0)	841 (68.2) 264 (21.3) 129 (10.5)	<0.0001
BMI; average ± SD (range)	24.7 ± 4.4 (16.7–42.0)	23.3 ± 3.6 (17.0–42.0)	25.4 ± 4.6 (15.8–43.8)	24.4 ± 4.3 (15.8–44.2)	<0.001
CHRUs; *n* (%)	97 (21.8)	74 (16.3)	20 (6.0)	191 (15.5)	<0.0001
Diabetes mellitus; *n* (%)	9 (2.0)	4 (0.9)	9 (2.7)	22 (1.8)	0.144
Cardiovascular diseases; *n* (%)	47 (10.5)	29 (6.4)	64 (19.2)	140 (11.4)	<0.0001
Respiratory diseases; *n* (%)	20 (4.5)	11 (2.4)	13 (3.9)	44 (3.6)	0.229
Immunodeficiency disorders; *n* (%)	28 (6.3)	24 (5.3)	17 (5.1)	69 (5.6)	0.728
solid or hematologic malignancies; *n* (%)	9 (2.0)	6 (1.3)	2 (0.6)	17 (1.4)	0.242

**Table 3:** Characteristics of the BI cases by occupational categories: vaccination status, duration, source of the infection and outcomes.

	Nurses (*n* = 446)	Doctors (*n* = 455)	Other HCWs (*n* = 333)	Total (*n* = 1234)	*p*-Value
Diagnosis of infection after vaccination dose; *n* (%) Vaccine dose n. 2 Vaccine dose n. 3	23 (5.2) 423 (94.8)	12 (2.6) 443 (97.4)	18 (5.4) 315 (94.6)	53 (4.3) 1181 (95.7)	0.100
Duration of the infection; average ± SD (range)	10.7 ± 3.8 (5–33)	11.1 ± 4.1 (5–41)	10.9 ± 3.9 (5–31)	10.9 ± 3.9 (5–41)	0.571
Source of the infection; *n* (%) Non-occupational Other HCWs Patient Not known	236 (52.9) 60 (13.5) 44 (9.9) 106 (23.7)	246 (54.1) 61 (13.4) 42 (9.2) 106 (23.3)	165 (49.6) 36 (10.8) 31 (9.3) 101 (30.3)	647 (52.4) 157 (12.7) 117 (9.5) 313 (25.4)	0.354
Reason for testing; *n* (%) close contact symptoms screening other	5 (1.1) 276 (61.9) 164 (36.8) 1 (0.2)	0 (0.0) 287 (63.1) 168 (36.9) 0 (0.0)	2 (0.6) 217 (65.2) 113 (33.9) 1 (0.3)	7 (0.5) 780 (63.2) 445 (36.1) 2 (0.2)	0.308
Symptoms; *n* (%) No symptoms Minor symptoms Major symptoms	7 (1.6) 434 (97.3) 5 (1.1)	8 (1.8) 443 (97.4) 4 (0.8)	11 (3.3) 319 (95.8) 3 (0.9)	26 (2.1) 1196 (96.9) 12 (1.0)	0.500
Duration of symptoms; average ± SD (range)	9.4 ± 5.0 (0–31)	9.0 ± 5.0 (0–46)	9.0 ± 5.3 (0–29)	9.2 ± 5.1 (0–46)	0.318
Hospitalization; *n* (%)	1 (0.2)	1 (0.2)	0 (0.0)	2 (0.2)	0.691
Hospitalization in ICU; *n* (%)	0 (0.0)	1 (0.2)	0 (0.0)	1 (0.1)	0.425
Persistence of symptoms after recovery from the infection; *n* (%)	81 (18.2)	40 (8.8)	55 (16.5)	176 (14.3)	<0.0001
Duration of symptoms after recovery from infection <15 days; *n* (%) ≥15 days; *n* (%)	80 (98.8) 1 (1.2)	39 (97.5) 1 (2.5)	54 (98.2) 1 (1.8)	173 (98.3) 3 (1.7)	0.877

**Table 4:** Characteristics of the BI cases: CLRUs vs. CHRUs.

	CLRUs (*n* = 1043)	CHRUs (*n* = 191)	Total (*n* = 1234)	*p*-Value
Diagnosis of infection after vaccination dose; *n* (%) Vaccine dose n. 2 Vaccine dose n. 3	43 (4.1) 1000 (95.9)	10 (5.2) 181 (94.8)	53 (4.3) 1181 (95.7)	0.547
Duration of the infection; average ± SD (range)	10.9 ± 3.9 (5–33)	11.0 ± 4.3 (5–41)	10.9 ± 3.9 (5–41)	0.796
Source of the infection; *n* (%) Non-occupational Other HCWs Patient Not known	528 (50.6) 137 (13.1) 98 (9.4) 280 (26.9)	119 (62.3) 20 (10.5) 19 (10.0) 33 (17.2)	647 (52.4) 157 (12.7) 117 (9.5) 313 (25.4)	0.012
Reason for testing; *n* (%) Close contact Symptoms Screening Other	7 (0.7) 666 (63.8) 368 (35.3) 2 (0.2)	0 (0.0) 114 (59.7) 77 (40.3) 0 (0.0)	7 (0.6) 780 (63.1) 445 (36.1) 2 (0.2)	0.358
Symptoms; *n* (%) no symptoms minor symptoms major symptoms	24 (2.3) 1007 (96.5) 12 (1.2)	0 (0.0) 189 (99.0) 2 (1.0)	26 (2.1) 1196 (96.9) 12 (1.0)	0.174
Duration of symptoms; average ± SD (range)	9.1 ± 5.0 (0–31)	9.6 ± 5.5 (0–46)	9.2 ± 5.1 (0–46)	0.356
Persistence of symptoms after recovery from the infection; *n* (%)	152 (14.6)	24 (12.6)	176 (14.3)	0.466
Duration of symptoms after recovery from infection; *n* (%) <15 days ≥15 days	149 (98.0) 3 (2.0)	24 (100.0) 0 (0.0)	173 (98.3) 3 (1.7)	0.488

## Data Availability

Data sharing will take place according to the guidelines established within the Orchestra project (www.orchestra-cohort.eu) (accessed on 29 September 2023).
